# Cellular nucleic acid-binding protein restricts SARS-CoV-2 by regulating interferon and disrupting RNA–protein condensates

**DOI:** 10.1073/pnas.2308355120

**Published:** 2023-11-14

**Authors:** Yongzhi Chen, Xuqiu Lei, Zhaozhao Jiang, Fiachra Humphries, Krishna Mohan Parsi, Nicholas J. Mustone, Irene Ramos, Tinaye Mutetwa, Ana Fernandez-Sesma, René Maehr, Daniel R. Caffrey, Katherine A. Fitzgerald

**Affiliations:** ^a^Division of Innate Immunity, Department of Medicine, University of Massachusetts Chan Medical School, Worcester, MA 01605; ^b^Program in Molecular Medicine, Diabetes Center of Excellence, University of Massachusetts Chan Medical School, Worcester, MA 01605; ^c^Department of Microbiology, Icahn School of Medicine at Mount Sinai, New York, NY 10029; ^d^Department of Neurology, Icahn School of Medicine at Mount Sinai, New York, NY 10029; ^e^Division of Infectious Diseases and Immunology, Department of Medicine, University of Massachusetts Chan Medical School, Worcester, MA 01605

**Keywords:** antiviral, RNA-binding protein, phase separation

## Abstract

A detailed understanding of the innate immune mechanisms involved in restricting SARS-CoV-2 infection and how the virus disrupts these processes could reveal new strategies to boost antiviral mechanisms and develop therapeutics for COVID-19. Here, we identify cellular nucleic acid-binding protein (CNBP) as a key host factor controlling SARS-CoV-2 infection. In response to RNA-sensing pathways, CNBP is phosphorylated and translocates from the cytosol to the nucleus where it binds to the interferon-β enhancer to initiate transcription. Because SARS-CoV-2 evades immune detection by the host’s RNA-sensing pathways, CNBP is largely retained in the cytosol where it restricts SARS-CoV-2 directly, leading to a battle between the host and SARS-CoV-2 that extends beyond antiviral immune signaling pathways. We further demonstrated that CNBP binds SARS-CoV-2 viral RNA directly and competes with the viral nucleocapsid protein to prevent viral RNA and nucleocapsid protein from forming liquid–liquid phase separation (LLPS) condensates critical for viral replication. Consequently, cells and animals lacking CNBP have higher viral loads, and CNBP-deficient mice succumb rapidly to infection. Altogether, these findings identify CNBP as a key antiviral factor for SARS-CoV-2, functioning both as a regulator of antiviral IFN gene expression and a cell-intrinsic restriction factor that disrupts LLPS to limit viral replication and spread. In addition, our studies also highlight viral condensates as important targets and strategies for the development of drugs to combat COVID-19.

## Significance

Understanding the host antiviral immune response to SARS-CoV-2 is critical to developing therapeutic strategies and gaining insights into why certain individuals suffer from severe infections. Our studies identify CNBP as a key host factor controlling SARS-CoV-2 infection. Antiviral agents blocking SARS-CoV-2 viral replication that complement vaccination are urgently needed to halt the current pandemic and prevent future outbreaks. Our foundational studies provide pivotal insights into the interactions of SARS-CoV-2 with the antiviral IFN system and reveal a mechanism that could be leveraged to develop therapeutic agents targeting LLPS.

The ongoing COVID-19 pandemic caused by severe acute respiratory syndrome coronavirus 2 (SARS-CoV-2) has placed an enormous burden on public health and the global economy, leading to >660 million infections and over 6.7 million deaths worldwide as of early 2023 (1–4). Infections with SARS-CoV-2 range from asymptomatic infection to severe and potentially fatal systemic inflammation, tissue damage, cytokine storm, and acute respiratory distress syndrome. A clearer understanding of how the immune system defends against SARS-CoV-2 is needed to determine the underlying causes of the variation in severity of COVID-19 and provide new opportunities for prevention and treatment (5, 6).

The innate immune system is the first line of host defense against SARS-CoV-2 (7). The antiviral interferon (IFN) system is an important component of the mammalian innate immune response and is activated when host pattern recognition receptors (PRRs) recognize and bind to pathogen-associated molecular patterns (PAMPs) of invading viruses (8–11). Host PRRs then activate transcription factors to induce type I IFN production. The activation of the antiviral IFN response contributes to SARS-CoV-2 symptoms and disease severity (12, 13). Moreover, IFN therapy has been suggested to be effective for SARS-CoV-2 patients (14, 15); however, the induction of type I IFNs is weak and delayed, and very low levels of type I IFNs are detected in the lungs or blood of infected patients compared to that seen with other viruses. In many individuals, this weak response is associated with increased disease severity (16, 17). In infected epithelial cells, the virus is poorly detected by innate immune sensors, indicating that SARS-CoV-2 efficiently counteracts the antiviral system. Indeed, increasing evidence demonstrates that SARS-CoV-2 is particularly adept at evading host innate immunity through deploying a range of countermeasures to subvert type I IFN responses to overcome innate antiviral defenses (12, 18–20). Thus, IFN-independent antiviral mechanisms are required to combat SARS-CoV-2 infection.

In addition to the type I IFN response, the host defends against RNA viruses using an arsenal of RNA-binding proteins (RBPs) that target viral RNA. These RBPs inhibit viral infection at different steps, ranging from the recognition of the invading viral RNA to the restriction of viral replication (21–24). Because SARS-CoV-2 is a positive-sense single-stranded (ss)RNA betacoronavirus in the *Coronaviridae* family, its viral RNAs play a central role during infection as they contain all the necessary information for an RNA virus to express its proteins, replicate, and spread. The ability of viral RNA to subvert cellular RBPs determines the outcome of a viral infection.

Another critical step in the lifecycle of RNA viruses is liquid–liquid phase separation (LLPS). LLPS is a process by which biomolecules, such as proteins or nucleic acids, condense into a dense phase that often resembles liquid droplets. Several viral processes incorporate phase separation, including viral replication and packaging (25, 26). During infection, LLPS serves as a scaffold for viral replication and promotes the assembly of machinery for viral production. For SARS-CoV-2, specific viral RNA sequences and structures play an important role in regulating nucleocapsid (N) protein LLPS (27–30).

Here, we demonstrated that CNBP is an antiviral protein that protects against SARS-CoV-2 infection using both IFN-dependent and independent mechanisms. CNBP is a DNA- and RNA-binding protein that is highly conserved across mammals and is involved in gene transcription and translation. Previously, we identified CNBP as a key signaling molecule activated downstream of RNA-sensing PRRs that control the transcription of type I IFNs to dsRNA and RNA viruses (31). CNBP is phosphorylated downstream of Toll-like receptors (TLRs) and RIG-I-like receptors (RLRs) by transforming growth factor β (TGFβ)-activated protein kinase 1 (TAK1). Phosphorylated CNBP translocates to the nucleus, where it binds the interferon-β (IFN-β) enhancer together with IFN-regulatory factor 3 (IRF-3) to turn on the transcription of type I IFNs and antiviral responses. Two independent groups reported an unbiased analysis of host proteins that bind to SARS-CoV-2 viral RNA (32, 33). In both studies, CNBP was the top SARS-CoV-2 genomic RNA-host binding protein identified and was demonstrated to have antiviral activity against SARS-CoV-2; however, the molecular mechanisms of its antiviral activity were not determined. Here, we show that CNBP inhibits SARS-CoV-2 replication in vitro and in vivo by regulating type I IFN; further, CNBP functions as a cell-intrinsic restriction factor that binds viral RNA and disrupts LLPS to limit viral replication and spread when SARS-CoV-2 evades the IFN system. These studies not only provide pivotal insights into the interactions of SARS-CoV-2 with the antiviral IFN system but also uncover a role for CNBP in restricting SARS-CoV-2.

## Results

### CNBP Inhibits SARS-CoV-2 Replication In Vitro by Regulating Type I IFN.

**Fig. 1.**
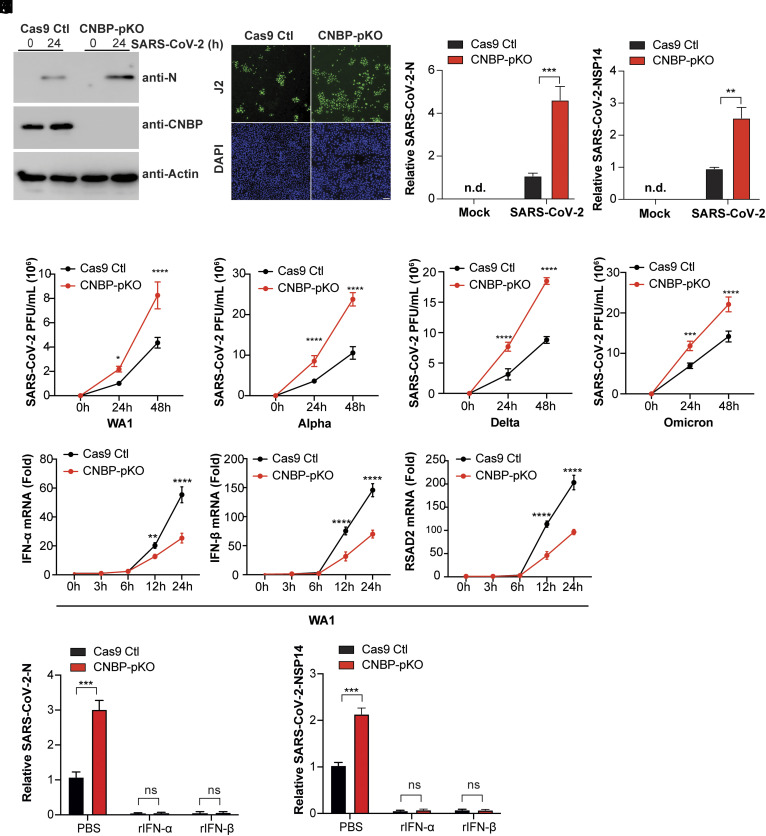
CNBP inhibits SARS-CoV-2 replication in vitro. (*A*–*D*) CNBP pKO and Cas9 control (Ctl) A549 cells were infected with the SARS-CoV-2 WA1 reference strain at an MOI of 0.01. At 24 h postinfection, western blotting with viral N protein (N) expression (*A*), immunofluorescence staining with anti-J2 antibody. Scale bars, 50 μm (*B*), and qPCR analysis of viral RNA levels of N (*C*) and NSP14 (*D*) were determined. (*E*–*H*) CNBP pKO and Cas9 Ctl A549 cells were infected with SARS-CoV-2 WA1 (*E*) or the alpha (*F*), delta (*G*), and omicron (*H*) variants, and viral titers in the supernatants were determined by plaque assays at the indicated time points. (*I*–*K*) Normalized RNA levels of IFN-α (*I*), IFN-β (*J*), and RSAD2 (*K*) in hACE2-A549 cells infected with SARS-CoV-2 WA1. (*L* and *M*) qRT-PCR analysis of SARS-CoV-2 mRNA expression in CNBP pKO and Cas9 control (Ctl) A549 cells pretreated with recombinant rIFNa or rIFNb. Error bars represent the SEM of triplicate biological replicates. All data are representative of three independent experiments. n.d., not detected; n.s., not significant; **P < 0.01; ***P < 0.001; ****P < 0.0001.

Our previous studies identified a role for CNBP in antiviral immunity to RNA viruses (31). As CNBP orthologs have undergone few amino acid changes throughout mammalian evolution (*SI Appendix*, Fig. S1*A*), we hypothesized that CNBP is highly conserved because of its essential antiviral function and examined its role in SARS-CoV-2 infection. Using A549-ACE2-expressing cells that are permissive to SARS-CoV-2 infection, we generated CNBP-deficient A549-ACE2 cells using CRISPR/Cas9 gene editing. We then infected these cells with the WA1 SARS-CoV-2 reference strain and found that CNBP-deficient cells (CNBP-pKO) had greater SARS-CoV-2 replication than wild-type (WT) cells. The levels of SARS-CoV-2 protein (assessed using anti-N antibodies) were higher in CNBP-deficient cells (Fig. 1*A*). As a readout of virus infection, we also monitored the accumulation of dsRNA using J2 antibody staining by immunofluorescence and found increased levels of J2 staining in CNBP-deficient cells (Fig. 1*B*). Similarly, these cells had higher N and NSP14 viral RNA levels (Fig. 1 *C* and *D*). To determine whether CNBP exhibited antiviral activity against a range of SARS-CoV-2 variants, we further tested the effect of knocking out CNBP on SARS-CoV-2 infection using the SARS-CoV-2 alpha, delta, and omicron variants. We observed higher viral titers in CNBP knockout (KO) cells infected with the SARS-CoV-2 strains (Fig. 1 *E*–*H*). In addition, we observed similar effects with HCoV-OC43 infection, a related betacoronavirus (*SI Appendix*, Fig. S1 *B* and *C*). To determine whether the role of CNBP is conserved in other cell types, we performed CNBP KO experiments in human Huh7.5 cells (a hepatocyte-derived cellular carcinoma cell line). Our results showed that SARS-CoV-2 replication was significantly increased in CNBP KO Huh7.5 cells (*SI Appendix*, Fig. S1 *D*–*F*). In addition to gene depletion studies, we examined SARS-CoV-2 replication in cells engineered to overexpress CNBP. A549-hACE2 cells overexpressing CNBP had reduced levels of viral RNA compared with vector control cells (*SI Appendix*, Fig. S1 *G*–*K*). To examine CNBP in a physiologically relevant cell system, we overexpressed CNBP in a three-dimensional lung alveolar cell system derived from human embryonic stem cells (hESCs). For these experiments, hESCs were differentiated into alveolar type II (iAT2) cells overexpressing CNBP and cultured in three dimensions at an air–liquid interface and infected with SARS-CoV-2. iAT2 cells overexpressing CNBP had reduced SARS-CoV-2 viral titers compared to parental cells (*SI Appendix*, Fig. S1 *L* and *M*). These knockout and overexpression studies indicate that the inhibitory effect of CNBP on SARS-CoV-2 replication was conserved across different variants and cell types.

As CNBP was previously reported to be essential for RNA virus-induced IFN production, we first examined the role of CNBP in IFN expression during SARS-CoV-2 infection. The quantitative PCR data confirmed that the levels of IFN-α and IFN-β in SARS-CoV-2-infected A549-ACE2 cells decreased in cells lacking CNBP (Fig. 1 *I* and *J*). We observed a similar trend for *RSAD2*, an IFN-stimulated gene (ISG) (Fig. 1*K*). To further confirm that the increased viral replication is due to reduced IFN-α or IFN-β, A549-hACE2 cells were pretreated with recombinant (r)IFN-α or rIFN-β and then infected with SARS-CoV-2 to detect viral replication. Administering exogenous rIFNa or rIFNb decreased viral replication in CNBP KO cells (Fig. 1 *L* and *M*). Together, these data indicate that CNBP plays a role in limiting the replication of SARS-CoV-2 and related coronaviruses in vitro by regulating type I IFN production.

### CNBP Inhibits SARS-CoV-2 Infection In Vivo.

**Fig. 2.**
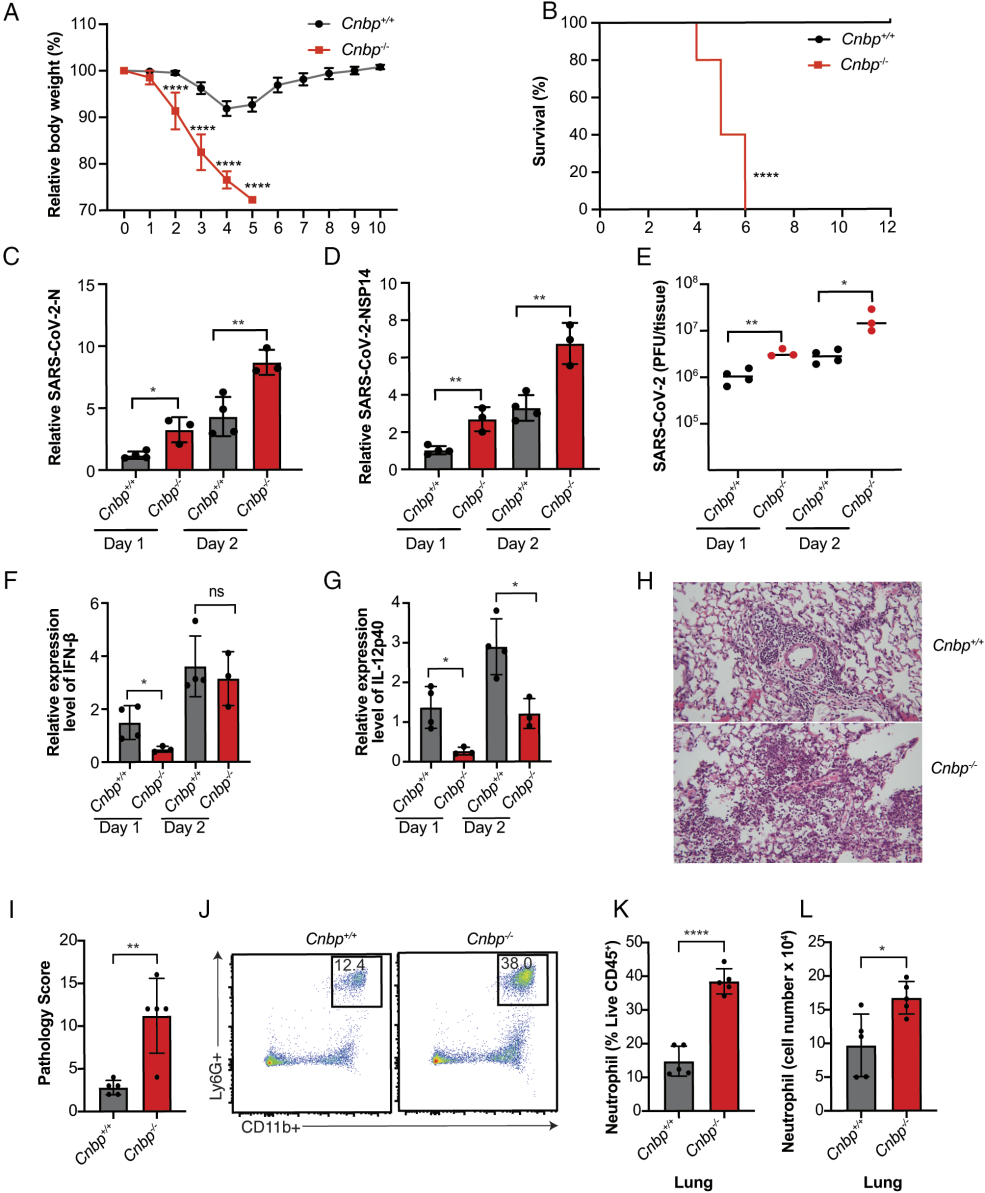
CNBP inhibits SARS-CoV-2 infection in vivo. (*A* and *B*) Weight loss (*A*) and survival (*B*) of WT and *Cnbp*^−/−^ mice intranasally infected with SARS-CoV-2 MA10 strain (1*10e5 PFUs). (*C* and *D*) WT and *Cnbp*^−/−^ mice were infected intranasally with the SARS-CoV-2 MA10 strain (1*10e5 PFUs). On days 1 and 2 postinfection (p.i.)., lungs were collected for qRT-PCR analysis of viral RNA levels of N (*C*) and NSP14 (*D*). (*E*) Viral lung titers of WT and *Cnbp*^−/−^ mice at 1 and 2 dpi. (*F* and *G*) Normalized mRNA levels of IFN-β (*F*) and IL12p40 (*G*) from lung samples of mice infected with SARS-CoV-2 MA10 strain. (*H* and *I*) Representative images (*H*) and pathology evaluation (*I*) of H&E stained lung sections from WT and *Cnbp*^−/−^ mice at 4 dpi of SARS-CoV-2 MA10. (*J*–*L*) Flow plots (*J*), percentage (*K*), and cell number (*L*) of neutrophils in the lung from WT and *Cnbp*^−/−^ mice at 4 dpi Each symbol represents an individual mouse; small horizontal lines indicate the mean. All data are representative of at least two to three independent experiments with similar results. n.s., not significant; *P < 0.05; **P < 0.01; ****P < 0.0001.

We next tested whether CNBP was important in restricting SARS-CoV-2 in vivo by infecting CNBP-deficient mice and WT littermate controls. We used a mouse-adapted SARS-CoV-2 MA10 variant (ic2019-nCoV MA10) that efficiently infects C57BL/6 mice (34). WT and CNBP-deficient mice were infected with MA10 (1 × 10^5^ PFUs) and monitored for weight loss and survival for 10 d. Wild-type animals exhibited transient weight loss (5 to 10%) after infection and recovered rapidly. In contrast, *Cnbp*^−/−^ mice lost weight rapidly and all succumbed to the infection within 6 d (Fig. 2 *A* and *B*). Furthermore, we monitored RNA levels and viral titers in the lungs at 1 or 2 days postinfection (dpi) and found that the levels of viral RNA or viral titers were higher in *Cnbp*^−/−^ mice compared to the WT littermate controls (Fig. 2 *C*–*E*). We also detected slightly higher viral RNA in the spleen, liver, and kidney of *Cnbp*^−/−^ mice than in WT mice, although the infection was still largely contained to the lung (*SI Appendix*, Fig. S2 *A* and *B*). Consistently, we detected reduced IFN-β and interleukin-12 p40 (IL12p40) mRNAs in *Cnbp*^−/−^ mice at early time points (Fig. 2 *F* and *G*); however, these KO mice had elevated TNF-α, IL-1β, and IL-10 mRNA, compared with WT mice (*SI Appendix*, Fig. S2 *C–E*). We also performed histopathological analysis on the lungs of mice infected with SARS-CoV-2 MA10. At 4 dpi, WT mice had evidence of alveolar septal thickening and mild inflammatory cell infiltration, whereas *Cnbp*^−/−^ mice showed severe alveolar septal thickening and infiltration of immune cells (Fig. 2 *H* and *I*). Flow cytometry demonstrated that neutrophil recruitment to the lungs was also elevated in *Cnbp*^−/−^ mice (Fig. 2 *J*–*L*). Collectively, these data demonstrate that CNBP plays a role in limiting SARS-CoV-2 replication in vivo.

### CNBP Phosphorylation and Translocation Are Weakly Activated by SARS-CoV-2.

**Fig. 3.**
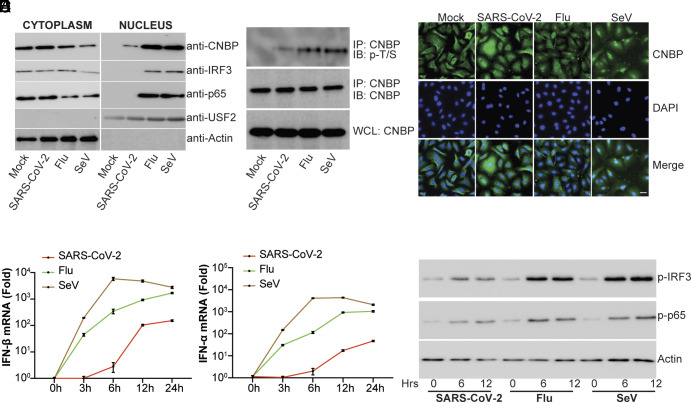
CNBP phosphorylation and translocation are weakly activated in SARS-CoV-2-infected cells. (*A*) Immunoblot analysis of nuclear translocation of CNBP, IRF3, or p65 in A549 cells infected with SARS-CoV-2, influenza virus (Flu), or SeV. Actin and upstream transcription factor 2 (USF2) were used as loading controls. (*B*) Endogenous CNBP protein was immunoprecipitated (IP) with anti-CNBP and immunoblotted (IB) with the anti-p-T/S to detect phosphorylation of CNBP after cells were infected with SARS-CoV-2, Flu, or SeV. (*C*) Localization of CNBP with or without SARS-CoV-2, Flu, or SeV infection as detected by immunofluorescence. (Scale bars, 20 μm.) (*D* and *E*) qPCR analysis of IFN-β mRNA (*D*) and IFN-α mRNA (*E*) induction by infection with SARS-CoV-2, Flu, or SeV at different time points. (*F*) Immunoblot analysis of p-IRF3 or p-p65 in whole-cell lysates of A549-hACE2 cells stimulated for various times with SARS-CoV-2, Flu, or SeV as indicated. Error bars represent SEM of triplicate biological replicates. All data are representative of three independent experiments.

Our previous studies demonstrated that endogenous CNBP is predominantly localized in the cytoplasm at steady state and, upon detection of a virus, becomes phosphorylated and translocates into the nucleus, where it induces IFN. In SARS-CoV-2-infected cells, however, the nuclear translocation and phosphorylation of CNBP are decreased compared to cells infected with influenza or Sendai virus (SeV) (Fig. 3 *A* and *B*). Further, immunofluorescence microscopy showed that CNBP was retained in the cytosol of SARS-CoV-2-infected cells (Fig. 3*C*). The decreased phosphorylation and translocation observed in A549-ACE2 cells infected with SARS-CoV-2 corresponded to a delayed IFN response that is decreased relative to that seen with either influenza or SeV (Fig. 3 *D* and *E*). Under these conditions, there was weak phosphorylation and translocation of IRF3 or p65, consistent with weak antiviral sensing in these cells (Fig. 3*F*), although SARS-CoV-2 is sensitive to IFN treatment as we observed markedly decreased viral RNA levels in the SARS-CoV-2-infected cells treated with rIFN-α, rIFN-β, and rIFN-γ (*SI Appendix*, Fig. S3 *A* and *B*). Together, these results demonstrate that CNBP is poorly activated in SARS-CoV-2-infected cells and suggest that SARS-CoV-2 is particularly adept at evading host innate immunity.

### CNBP Suppresses SARS-CoV-2 Replication through an IFN-Independent Mechanism.

**Fig. 4.**
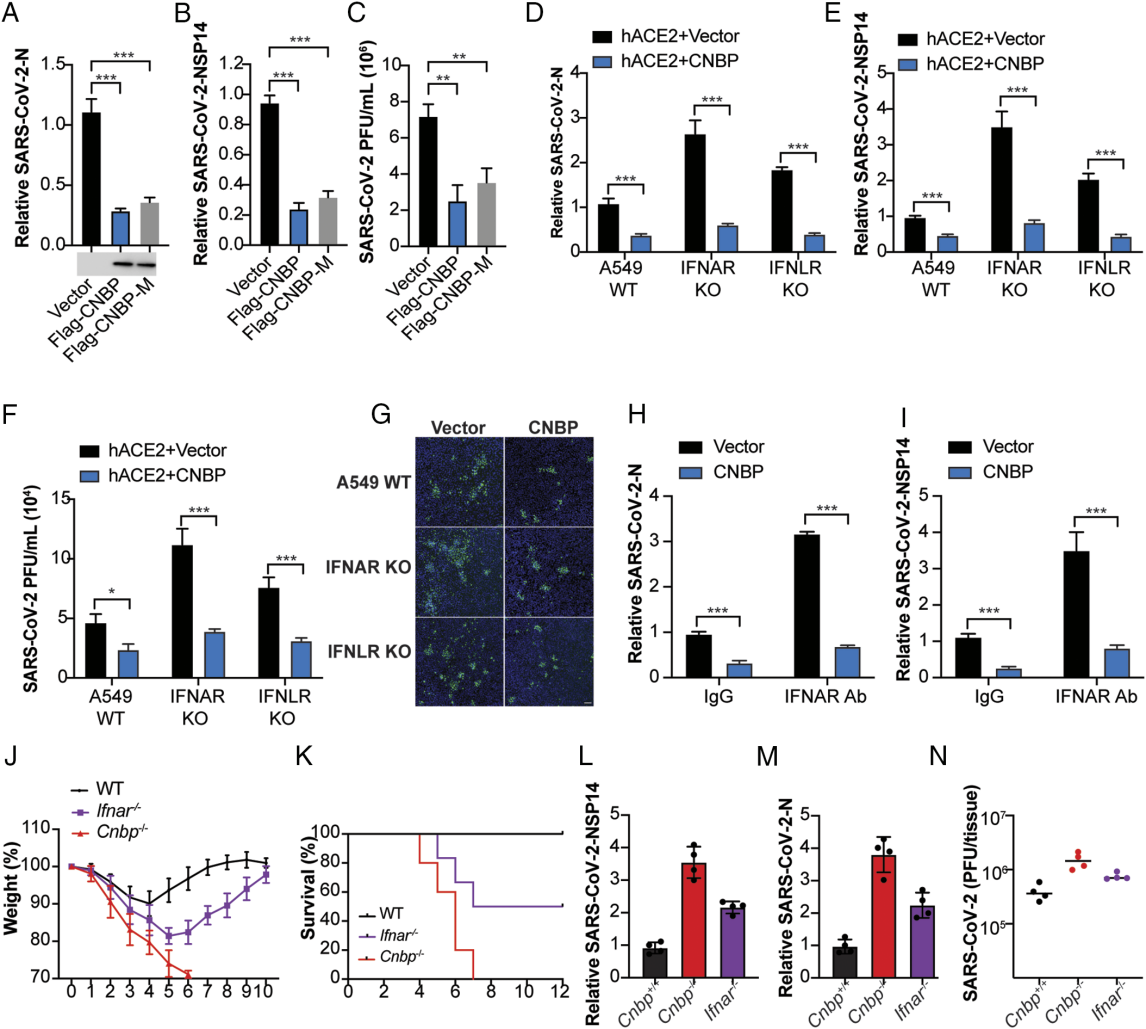
CNBP suppresses SARS-CoV-2 replication through an IFN-independent mechanism. (*A*–*C*) Normalized SARS-CoV-2 RNA levels of N (*A*) and NSP14 (*B*), as well as the SARS-CoV-2 titer (*C*) in hACE2-A549 cells transfected with Flag-CNBP or Flag-CNBP mutant plasmid and infected with SARS-CoV-2. (*D*–*G*) IFNAR KO, IFNLR KO, and Cas9 Ctl A549 cells cotransfected with a hACE2 plasmid with Flag-CNBP or Flag-CNBP-M were infected with SARS-CoV-2 at an MOI of 0.1. At 24 h postinfection, viral RNA levels of N (*D*) and NSP14 (*E*) were assessed by qPCR, and viral titers (*F*) in the supernatants were determined by plaque assay and immunofluorescence staining with anti-J2 antibody. Scale bars, 50 μm (*G*). (*H* and *I*) qRT-PCR analysis of SARS-CoV-2 gRNA expression of N (*H*) and NSP14 (*I*) in hACE2-A549 cells overexpressing CNBP treated with the neutralizing antibody anti-IFNAR. (*J* and *K*) Weight loss (*J*) and survival (*K*) of WT, *Cnbp*^−/−^, and *Ifnar*^−/−^ mice intranasally infected with SARS-CoV-2 MA10 strain (1*10e5 PFUs). (*L* and *M*) WT, *Cnbp*^−/−^, and *Ifnar*^−/−^ mice were infected intranasally with SARS-CoV-2 MA10 strain (1*10e5 PFUs), and on day 2 postinfection (p.i.), the lungs were collected for qRT-PCR analysis of viral RNA levels of N (*L*) and NSP14 (*M*). (*N*). Viral lung titers of WT, *Cnbp*^−/−^ and *Ifnar*^−/−^ mice at 2 dpi Error bars represent SEM of triplicate biological replicates. All data are representative of three independent experiments. *P < 0.05; **P < 0.01; ***P < 0.001.

The weak induction of IFN in SARS-CoV-2-infected cells prompted us to examine whether CNBP might also limit SARS-CoV-2 infection via an IFN-independent mechanism. Previous work from our lab and others demonstrated that CNBP is phosphorylated by TAK1 kinase, which in turn controls its nuclear translocation (31, 35). We took advantage of a phosphorylation-defective T173/177A CNBP mutant (CNBP-M) that is retained in the cytosol and fails to regulate the type I IFN response. We tested whether CNBP-M could still restrict SARS-CoV-2 replication in transfected A549-ACE2 cells. To this end, we transfected the WT and CNBP-M cells and monitored SARS-CoV-2 infection (Fig. 4 *A*–*C*). The CNBP-M was just as effective as the WT in blocking infection, suggesting that CNBP still inhibits SARS-CoV-2 infection independent of its role as a signaling molecule controlling type I IFN gene expression. Consistent with this finding, overexpression of CNBP still blocked SARS-CoV-2 replication in IFN α/β receptor (IFNAR) KO A549 ACE2 cells (Fig. 4 *D*–*G*). Similar results were obtained with A549-ACE2 cells lacking the IFNλ receptor (IFNLR). Further, overexpression of CNBP blocked SARS-CoV-2 replication in cells treated with an anti-IFNAR antibody (Fig. 4 *H* and *I*). Consistently, there was no significant difference in viral loads between the IFNAR-blocking Ab treatment and IgG treatment conditions in CNBP KO cells; however, the CNBP KO cells were more susceptible than WT cells treated with IFNAR-blocking Ab, further supporting a model whereby CNBP has IFN-independent antiviral functions on SARS-CoV-2 (*SI Appendix*, Fig. S4 *A* and *B*). Similar results were obtained using the human coronavirus OC43 (HCoV-OC43) (*SI Appendix*, Fig. S4 *C–F*). These results indicate that CNBP also inhibits SARS-CoV-2 replication through IFN-independent mechanisms, and this mechanism is conserved across multiple coronaviruses.

In support of our in vitro findings, *Cnbp*^−/−^ mice were more susceptible to SARS-CoV-2 infection than *Ifnar*^−/−^ mice. While 100% of the *Cnbp*^−/−^ mice succumbed to SARS-CoV-2 infection, only ~50% of the *Ifnar*^−/−^ mice succumbed to the infection at this dose (Fig. 4 *J* and *K*). Consistently, the levels of viral RNA or viral titers were higher in *Cnbp*^−/−^ mice compared to *Ifnar*^−/−^ mice (Fig. 4 *L*–*N*). The more pronounced susceptibility of *Cnbp*^−/−^ mice relative to *Ifnar*^−/−^ mice provides additional support for IFN-independent functions of CNBP in restricting SARS-CoV-2.

### CNBP Binds to SARS-CoV-2 Viral RNA through Its RGG and Linker Region.

**Fig. 5.**
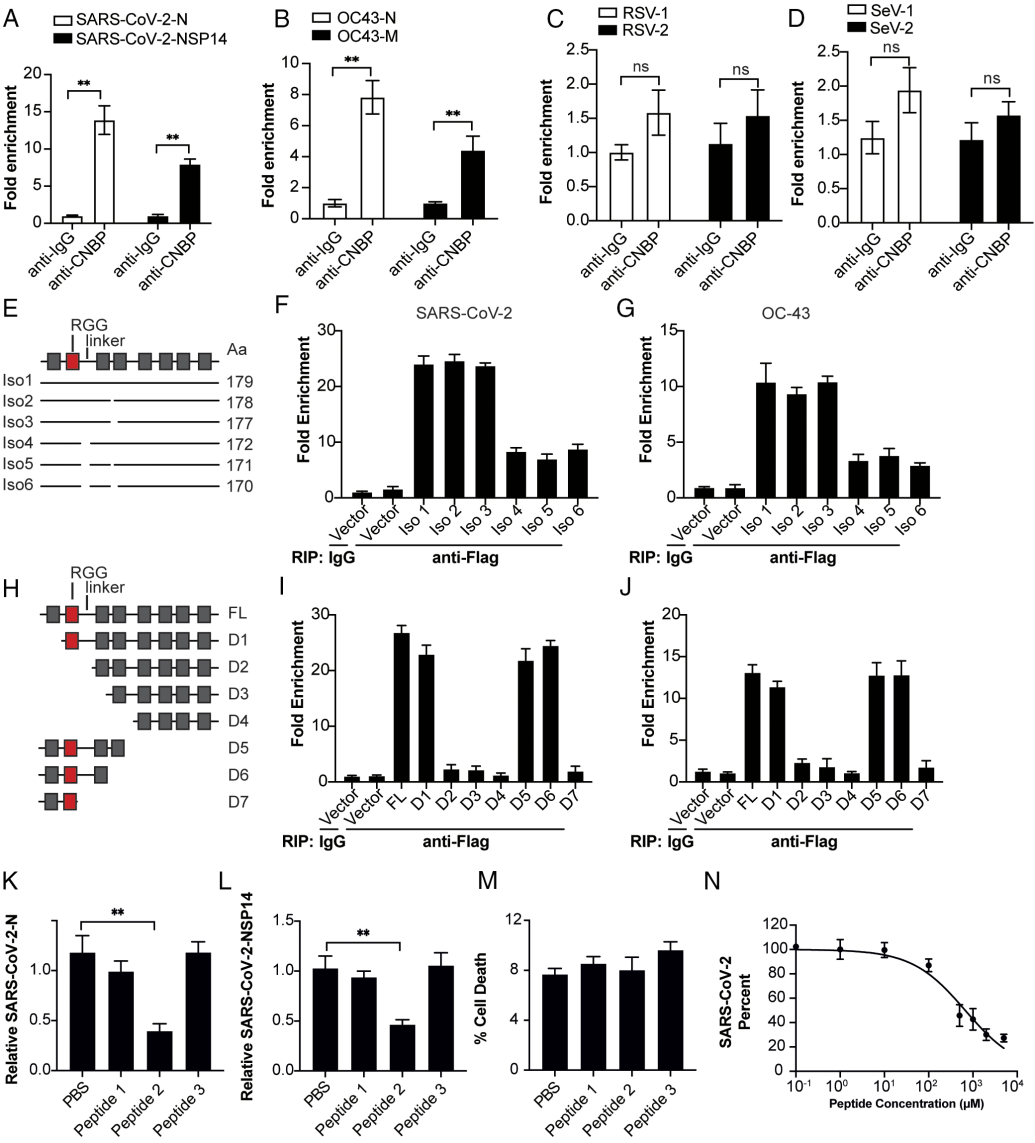
CNBP binds SARS-CoV-2 viral RNA. (*A*–*D*) RIP assay with hACE2-A549 cell lysates prepared after 24 h of infection with SARS-CoV-2, OC43 virus, respiratory syncytial virus (RSV), or Sendai virus (SeV) using anti-CNBP or control immunoglobulin. Immunoprecipitated SARS-CoV-2 RNA (*A*), OC43 virus RNA (*B*), RSV RNA (*C*), or SeV RNA (*D*) was quantified by qRT-PCR. (*E*) Comparison of the six different human CNBP isoforms. The horizontal lines indicate regions in each isoform. The RGG and zinc fingers are shown as red and gray boxes respectively. (*F* and *G*) Analysis of the RNA binding activity of the six CNBP isoforms with SARS-CoV-2 RNA (*F*) or OC43 virus RNA (*G*). (*H*) Map of the functional regions of full-length CNBP and deletion constructs. (*I* and *J*) Analysis of the RNA binding activity of the CNBP deletion constructs with SARS-CoV-2 RNA (*I*) or OC43 virus RNA (*J*). (*K* and *L*) Analysis of the antiviral function of the CNBP peptides on SARS-CoV-2. (*M*) Lactate dehydrogenase (LDH) assay for cell death in the presence of the peptides. (*N*) The IC_50_ values for Peptide 2 are calculated from the cell-based antiviral activity data. Error bars represent SEM of triplicate biological replicates. All data are representative of three independent experiments. n.s., not significant; **P < 0.01.

We next wanted to understand how CNBP limits SARS-CoV-2 infection through an IFN-independent mechanism. In two separate studies that used an unbiased analysis to identify host proteins that bind to SARS-CoV-2 genomic RNA, CNBP was the top hit (32, 33). We, therefore, hypothesized that CNBP inhibits SARS-CoV-2 infection by binding viral RNA directly. First, we demonstrated that CNBP directly binds SARS-CoV-2 viral RNA by performing RNA immunoprecipitation (RIP) followed by qPCR to quantify levels of the nucleocapsid (N) and nonstructural protein 14 (NSP14) viral RNAs. We found SARS-CoV-2 viral RNA enriched in the CNBP pull-downs (Fig. 5*A*). CNBP could also bind RNA from HCoV-OC43 but not respiratory syncytial virus (RSV) or SeV (Fig. 5 *B*–*D*).

Human CNBP encodes six splice isoforms in A549 cells that change the composition of its nucleic acid-binding domains and potentially modify target specificity (Fig. 5*E* and *SI Appendix*, Fig. S5*A*). Thus, we investigated the RNA-binding capability of the six CNBP isoforms with viral RNA during SARS-CoV-2 infection. Using RIP-qPCR, we showed that CNBP isoforms 1 to 3 have higher binding affinity compared with isoforms 4 to 6, suggesting that the deletion of seven amino acids (GFTSDRG) at the N-terminal end of the linker region between the first and second zinc finger was important for the binding with viral RNA (Fig. 5 *F* and *G*). We next investigated whether differences in the RG-rich domain could influence antiviral activity by examining the antiviral activities of the six CNBP isoforms. The results showed that the antiviral activities of CNBP isoforms 1 to 3 were higher than CNBP isoforms 4 to 6, indicating that the RNA-binding capability correlates with antiviral activity (*SI Appendix*, Fig. S5 *B* and *C*). In addition, we evaluated the role of the six CNBP isoforms in regulating IFN response with the IFN-β and IFN-α reporter assays. Notably, we demonstrated that all six isoforms could induce the IFN-β and IFN-α reporter activation at comparable levels (*SI Appendix*, Fig. S5 *D* and *E*). We next expressed the six CNBP isoforms together with IRF3 or IRF7, revealing that all six CNBP isoforms strongly synergized with IRF3 or IRF7 to activate the Ifn-β and Ifn-α promoter (*SI Appendix*, Fig. S5 *F* and *G*). Collectively, these results suggest that the antiviral activity of CNBP correlates with its RNA-binding capability and is independent of its role in regulating type I IFN.

To further define which domain of CNBP might be responsible for RNA binding, we generated multiple deletion mutants (Fig. 5*H*). We transfected expression plasmids encoding these domain deletion mutants with SARS-CoV-2 infection and performed RIP-qPCR. We found that all the deletion mutants lacking the RGG and linker region lost their RNA-binding activity and antiviral activity against SARS-CoV-2 (Fig. 5 *I* and *J* and *SI Appendix*, Fig. S5 *H* and *I*). To further confirm that CNBP directly binds SARS-CoV-2 viral RNA, a microscale thermophoresis (MST) study was performed using recombinant full-length CNBP or CNBP mutants (*SI Appendix*, Fig. S5*J*). The SARS-CoV-2 genome RNA bound to full-length CNBP protein with an equilibrium dissociation constant (Kd) of 29.66 ng/µL, and the binding affinity of the CNBP mutant D6 was comparable with that of the full-length CNBP. In addition, CNBP mutant D3 lacking the RGG and linker regions showed almost no binding to SARS-CoV-2 genome RNA, further demonstrating that the RGG and linker region of CNBP play an important role in binding viral RNA. Intermediate molecular weight peptides, which mimic the RNA-binding proteins, can provide much greater surface area and, therefore, have greater potential to form high affinity and specific complexes to inhibit protein-RNA interactions. Thus, we designed and chemically synthesized small peptides targeting the RGG and linker region (*SI Appendix*, Fig. S5*K*). These peptides were linked to the HIV TAT protein transduction sequence to facilitate cellular uptake. As shown in Fig. 5 *K*–*N*, the presence of peptide 2 in A549 cells substantially inhibited SARS-CoV-2 replication with an IC_50_ value of 669 µM, while there was no antiviral effect on RSV or SeV (*SI Appendix*, Fig. S5 *L* and *M*), suggesting that a peptide mimicking the RGG and linker region of CNBP could restrict SARS-CoV-2 directly. Altogether, our results demonstrated that the RGG and linker region of CNBP play an important role in binding viral RNA leading to the inhibition of SARS-CoV-2 replication independent of IFN regulation.

### CNBP Binds to SARS-CoV-2 Viral RNA and Competes with the SARS-CoV-2 N Protein.

**Fig. 6.**
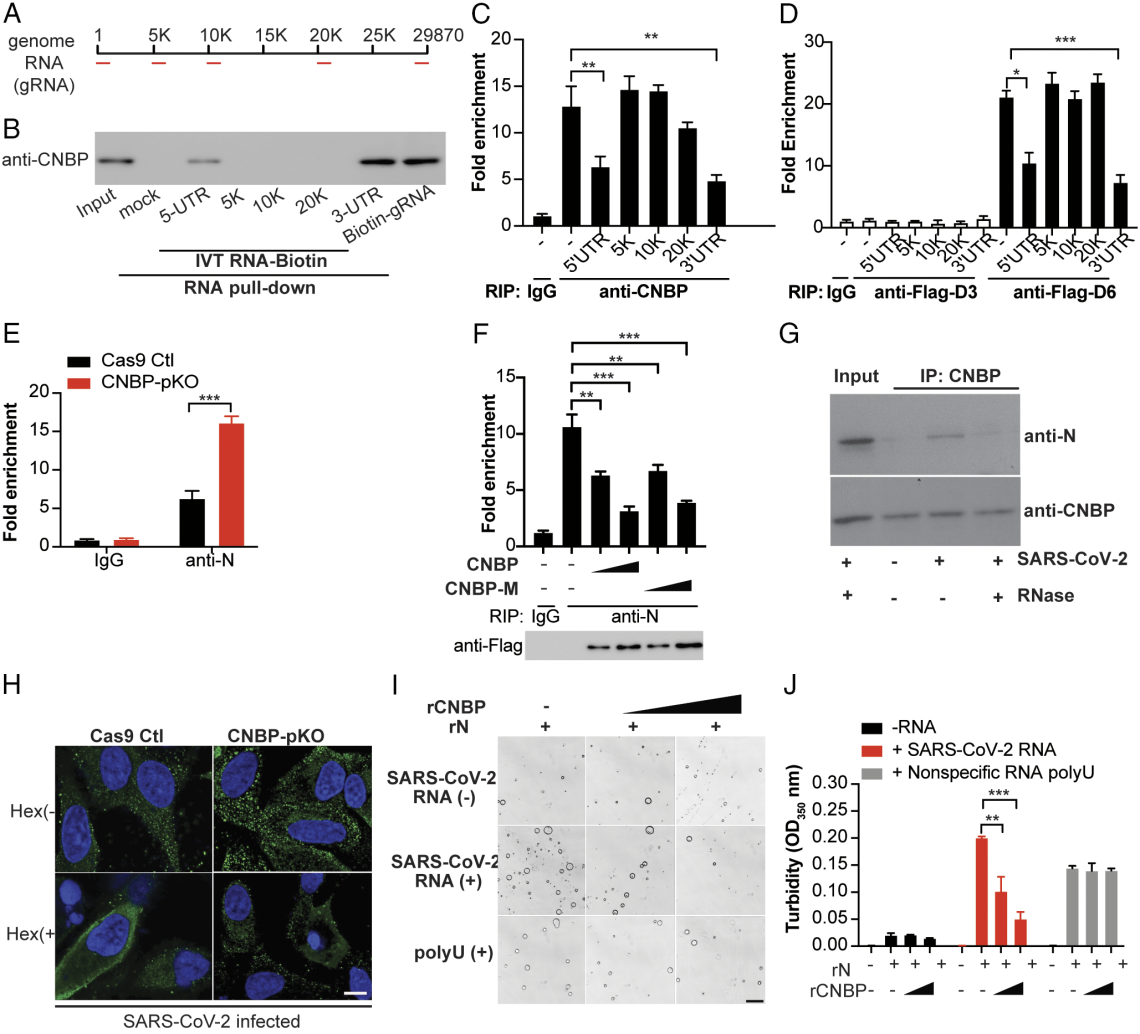
CNBP disrupts viral RNA-induced nucleocapsid protein condensates. (*A*) The map of the SARS-CoV-2 genomic RNA (gRNA) and in vitro transcription of the 5′ UTR, 5K, 10K, 20K, and 3′ UTR RNA fragments. (*B*) RNA pull-down assay showing the binding activity of SARS-CoV-2 RNA genome or in vitro-transcribed (IVT) RNAs to CNBP. (*C*) RIP assay and RT-qPCR analysis of the binding activity of CNBP with SARS-CoV-2 genomic RNA in the presence of the indicated IVT RNAs. (*D*) RIP assay and qRT-PCR analysis of the binding activity of Flag-CNBP deletions with SARS-CoV-2 genomic RNA in the presence of the indicated IVT RNAs. (*E*) RIP assay with A549 WT or CNBP pKO cell lysates prepared after 24 h of infection with SARS-CoV-2 by using anti-N. The immunoprecipitated SARS-CoV-2 positive-strand RNA was quantified by qRT-PCR. (*F*) CNBP pKO transfected with CNBP and CNBP-M, cell lysates were prepared after 24 h of infection with SARS-CoV-2, the interaction of SARS-CoV-2 positive-strand RNA with N was analyzed by RIP assay and qRT–PCR analysis as described in *C*. (*G*) Coimmunoprecipitation of CNBP and N in SARS-CoV-2-infected cell lysates treated with or without RNase. (*H*) Increased N puncta are formed in CNBP pKO cells compared with Cas9 Ctl hACE2-A549 cells infected with SARS-CoV-2 and disrupted by treating cells with 1,6-hexanediol. Scale bars, 10 μm (*I*) N protein LLPS were observed using a bright field confocal microscope and could be disrupted by the addition of rCNBP. Scale bars, 10 μm. (*J*) The turbidity of each sample was measured by absorbance at 350 nm. Error bars represent SEM of triplicate biological replicates. All data are representative of three independent experiments. *P < 0.05; **P < 0.01; ***P < 0.001.

To further define how CNBP binds SARS-CoV-2 viral RNA to inhibit replication, we generated biotin-labeled RNAs corresponding to the 5′ UTR, 3′ UTR, and three internal regions by in vitro transcription (IVT) and used these in pull-down experiments to map the region(s) of SARS-CoV-2 genomic RNA bound by CNBP. CNBP was enriched in the streptavidin pull-downs using both the 5′ UTR and 3′ UTR RNA fragments but not by the RNA fragments corresponding to internal regions of the genomic RNA (Fig. 6 *A* and *B*). We also performed the anti-CNBP RIP-qPCR experiments in infected cells and showed that endogenous CNBP binding to SARS-CoV-2 genomic RNA was reduced by incubating these pull-down reactions with IVT RNAs corresponding to the 5′ UTR and 3′ UTR but not by IVT RNAs from other regions of the genomic RNA (Fig. 6*C*). Moreover, RGG and the linker region of CNBP binding with viral RNA were also reduced by the 5′ UTR and 3′ UTR IVT RNAs, further confirming that CNBP could bind the 5′ UTR and 3′ UTR of viral RNA through the RGG and linker region (Fig. 6*D*).

The SARS-CoV-2 N protein is an RBP that plays a critical role in viral genome packaging and virion assembly. We speculated that CNBP might compete with the N protein for viral RNA. We confirmed viral RNA binding to the N protein by RIP-qPCR. Anti-N pull-downs demonstrated that N protein bound to viral RNAs in infected cells and N protein binding to RNA was elevated in cells lacking CNBP (Fig. 6*E*). Further, overexpression of CNBP or CNBP-M blocked the binding of the N protein to viral RNA in a dose-dependent manner (Fig. 6*F*). We could also detect N protein associated with CNBP during SARS-CoV-2 infection; however, the interaction between CNBP and SARS-CoV-2 N was sensitive to RNase digestion, suggesting that CNBP and SARS-CoV-2 N form a complex in the presence of viral RNA (Fig. 6*G*).

### CNBP Disrupts the LLPS Process of SARS-CoV-2.

Recently, several independent groups have reported that the N protein can undergo LLPS in the presence of viral genomic RNA, and the formation of these RNA–protein condensates increases the efficiency of viral RNA transcription and assembly of virions (36–39). The 5′ UTR and 3′ UTR are important in the formation of these RNA-N condensates (28, 29). We confirmed that N protein forms condensates in the presence of increasing concentrations of viral RNA, and the N-RNA condensates were dissolved by 5% 1,6-hexanediol, an organic solvent known to disrupt a wide range of biomolecular condensates (*SI Appendix*, Fig. S6*A*). A549-ACE2 cells showed the formation of N protein puncta after SARS-CoV-2 infection and the formation of these puncta was enhanced in CNBP-deficient cells (Fig. 6*H*). These puncta could be disrupted by treating cells with 1,6-hexanediol to disrupt condensates. The high level of N protein puncta in CNBP-deficient cells prompted us to test whether CNBP modulates the LLPS of N protein in vitro. As expected, CNBP itself failed to undergo LLPS (*SI Appendix*, Fig. S6 *B* and *C*). The N protein in the presence of viral RNA formed droplets, and recombinant CNBP inhibited the formation of these droplets—both the size and number of droplets decreased (Fig. 6*I*). The suppressive effect of CNBP was dose-dependent in this assay, as shown by quantifying the turbidity at 350 nm. Interestingly, the non-specific polyU homopolymer RNA also induced LLPS of N protein; however, these condensates were not impacted by CNBP (Fig. 6 *I* and *J*). Collectively, these data demonstrate that the SARS-CoV-2 N protein undergoes RNA-induced LLPS, and this process is disrupted by CNBP.

## Discussion

Patients with genetic mutations in antiviral genes that control type I IFN production suffer from life-threatening COVID-19 disease (16, 40). Further, autoantibodies that neutralize type I IFNs have also been identified in patients and correlated with more severe COVID-19 disease (41). Collectively, these observations highlight the important role innate antiviral responses play in curbing the replication of SARS-CoV-2. Here, we identify CNBP as a key host factor controlling SARS-CoV-2 infection. Consistent with its role in other RNA virus infections, CNBP coordinates signaling events that couple RNA sensing to type I IFN gene transcription. Cells lacking CNBP or receptors for type I or type III IFNs have elevated viral loads, and animals lacking CNBP are more susceptible to virus infection than their wild-type counterparts.

In response to the formidable antiviral defenses mounted by the host’s innate immune system, viruses have evolved countermeasures to subvert type I IFN responses (7, 42). SARS-CoV-2 is particularly adept at evading host innate immunity. Consequently, very low levels of type I IFNs are detected in the lungs or blood of infected patients compared to that seen with other viruses (43, 44). Indeed, our in vitro data also demonstrated that SARS-CoV-2 induces weak and delayed type I IFNs and ISGs in infected cells compared with other viruses. We consistently observed weak nuclear translocation and phosphorylation of IRF3, p65, and CNBP, suggesting that CNBP is poorly activated in SARS-CoV-2-infected cells, likely due to a failure of RNA sensors to appropriately recognize the virus and induce downstream signaling.

The host defense system against SARS-CoV-2 extends beyond antiviral immune signaling pathways. Due to the limited RNA sensing and immune evasion in SARS-CoV-2-infected cells, CNBP was retained in the cytosol in which SARS-CoV-2 replicates; however, it could still restrict SARS-CoV-2 replication in a cell-intrinsic manner. The association of the SARS-CoV-2 N protein with viral genomic RNA to generate higher-order RNA–protein complexes through LLPS is a key step in the replication of SARS-CoV-2, serving to concentrate RNA and proteins during virion assembly. CNBP targets this essential step by disrupting the phase separation that occurs with viral RNA and the N protein. Mechanistically, CNBP binds SARS-CoV-2 viral genomic RNA and precludes the N protein from forming condensates. CNBP binds the 5′ UTR and 3′ UTR, and these regions are important for the LLPS observed with N protein and viral RNAs. Our data showed that CNBP could also bind RNA from HCoV-OC43 but not respiratory syncytial virus (RSV) or SeV, suggesting that CNBP may bind specifically to positive-sense viruses. However, further research is needed to determine whether the RNA sequence, secondary structure, or specific binding motifs affect the specificity of CNBP for SARS-CoV-2 RNA. Thus, our findings demonstrate that CNBP disrupts the LLPS of the N protein and highlight that the LLPS step mediated by the N protein is a promising therapeutic target during SARS-CoV-2 infection. Indeed, several small molecules have been reported to inhibit viral replication by targeting the LLPS of the viral N (28, 45, 46). To our knowledge, CNBP is the first host factor that impacts viral replication by targeting viral-specific RNA sequences required for LLPS, revealing a host-directed antiviral strategy.

Recent work has highlighted how the N-RNA condensates contribute to viral transcription, replication, and immune evasion by targeting the mitochondrial antiviral-signaling protein (MAVS) as a mechanism to disrupt type I IFN signaling (47). Further, SARS-CoV-2 N LLPS facilitates NF-κB hyper-activation and inflammation through regulation of TAK1 and IκB kinase (IKK) (48). Our results also demonstrated that CNBP positively regulates type I IFN expression during RNA virus infection. Whether the disruption of the N protein LLPS by CNBP could restore innate antiviral immunity at the level of MAVS warrants further study. Consistent with CNBP’s ability to disrupt LLPS condensates and its role in controlling type I IFNs, we observed a marked susceptibility of CNBP-deficient mice to SARS-CoV-2 infection. The impact of CNBP deficiency was greater than that seen in IFNAR-deficient mice, underscoring the dual function of CNBP. The broad antiviral function of CNBP on RNA viruses depends on its activation and nuclear translocation to activate IFNs. Like other RNA viruses, SARS-CoV-2 could also be inhibited by CNBP-mediated IFN induction. However, due to the limited RNA sensing and immune evasion of RNA-sensing pathways in SARS-CoV-2-infected cells, CNBP is largely retained in the cytosol where it restricts SARS-CoV-2 through an IFN-independent mechanism. The temporal relationship between these two functions is still not clearly defined. One possibility is that they occur simultaneously, and the relative contribution of these two phenotypes may also depend on other factors, including the viral load, the cellular context, and the kinetics of the antiviral response. Further research is needed to dissect the precise molecular mechanisms underlying these dual functions and to determine how they are coordinated in SARS-CoV-2 infected cells. Additionally, the dynamic regulation and subcellular localization of CNBP in different cell types and under various conditions may provide additional insights into its roles in antiviral immunity.

A detailed understanding of the molecular mechanisms involved in restricting SARS-CoV-2 infection and how SARS-CoV-2 attempts to disrupt these mechanisms could reveal new therapeutic opportunities to boost antiviral mechanisms and clear SARS-CoV-2. Our results demonstrate that peptide mimics of CNBP could be optimized as an effective antiviral strategy. Altogether, our findings underscore the importance of CNBP during SARS-CoV-2 infection, highlighting the importance of this factor as a regulator of type I IFNs and as a cell-intrinsic restriction factor. The identification of distinct functional outcomes of CNBP, depending on its cellular location, provides important insights that could be leveraged to improve the outcome of host interactions with this potentially deadly pathogen. In addition, our studies also highlight targeting viral condensates as important targets and strategies for the development of drugs to combat COVID-19.

## Materials and Methods

### Biosafety.

All study protocols were reviewed and approved by the Environmental Health and Safety and Institutional Review Board at the University of Massachusetts Chan Medical School (UMass Chan) prior to study initiation. All experiments with SARS-CoV-2 were performed in a biosafety level 3 (BSL3) laboratory by personnel equipped with powered air-purifying respirators.

### Viruses.

Vero E6 cells were infected with the USA-WA1/2020 (NR-52281; BEI Resources) or the mouse-adapted MA10 variant of SARS-CoV-2 (in the isolate USA-WA1/2020 backbone) Infectious Clone (ic2019-nCoV MA10) from American Type Culture Collection (ATCC). Supernatants were centrifuged at 450 *g* for 10 min and aliquoted and stored at −80 °C. HCoV-OC43 was obtained from William M. McDougall (UMass Chan), RSV was obtained from Robert W. Finberg (UMass Chan), SeV (Cantell strain) and human influenza A/PR/8/34 (H1N1) were purchased from Charles River Laboratories. Virus titer was determined by a TCID_50_ assay in Vero E6 cells. For the purification of genomic SARS-CoV-2 RNA (gRNA), the supernatant from Vero cells infected with SARS-CoV-2 was lysed in TRIzol LS, and viral RNA was extracted from TRIzol using chloroform extraction.

### Human Pluripotent Stem Cell–Derived Type II Pneumocytes (iAT2).

Human pluripotent stem cell–derived type II pneumocytes (iAT2) were derived and differentiated from H1 human embryonic stem cells as previously described (49), and CNBP was transduced into iAT2 cells by lentivirus infection. After 2 wk of iAT2-ALI culture maturation, cells were used for SARS-CoV-2 infection experiments.

### Coimmunoprecipitation and Western Blot Analysis.

Cell lysis and immunoblot analysis were performed as described previously (31).

### Microscale Thermophoresis Assay.

The binding between CNBP or the CNBP mutant proteins and SARS-CoV-2 genome RNA was measured by MST assays. Briefly, purified CNBP or CNBP mutant proteins were fluorescently labeled according to the manufacturer’s procedure and kept in MST Buffer. RED-Tris-NTA second-generation dye (The Monolith His-Tag Labeling Kit RED-Tris-NTA 2nd Generation kit) was added, mixed, and incubated for 30 min at room temperature. The labeled proteins were mixed with each of the RNAs at 16 different serially diluted concentrations. The samples were then loaded into standard-treated capillaries (NanoTemper Technologies) and measured at 60% LED power, and high MST power. NanoTemper Analysis software was used to analyze the data, and the final plots were made using GraphPad Prism 9.0.

### In Vitro Phase Separation Assays.

Phase separation of NP [in 5 mM HEPES (pH 7.5), 100 mM NaCl] was induced by adding SARS-CoV-2 genomic RNA with increasing concentrations of CNBP protein. Samples were mixed and then immediately transferred onto microscope glass slides. Condensates were imaged within 10 to 20 min or as indicated in the experiment.

### Turbidity Measurements.

Turbidity was used to evaluate the phase separation of SARS-CoV-2 N protein at different conditions determined using a NanoDrop spectrophotometer. Increasing concentrations of CNBP were added immediately before the experiments, followed by thorough pipetting and measurement of turbidity by absorbance at 350 nm. Average turbidity values were derived from measurements of three independent, freshly prepared samples.

### RNA Immunoprecipitation (RIP)-qPCR.

Human ACE2-A549 cells were infected with SARS-CoV-2 (MOI = 1) for 24 h, and then, the cells were fixed using 4% paraformaldehyde (PFA) for 15 min. Cell lysates were immunoprecipitated with IgG, anti-CNBP, or anti-NP and incubated with protein G beads in a cold room overnight. The bead-bound immunoprecipitants were washed 3 times with lysis buffer, and the protein and RNA complexes were eluted with TE buffer. The RNA was extracted using TRIzol reagent before real-time PCR analysis for SARS-CoV-2 or OC43 RNA.

### Immunofluorescence.

Cells were fixed using 4% PFA for 30 min. After two PBS washes, cells were permeabilized with 0.2% Triton X-100/PBS before incubation with primary antibodies for 2 h at room temperature. Cells were washed in PBS, followed by incubation with secondary antibodies. Nuclei were stained with DAPI.

### In Vitro Transcription RNA Assay.

The full RNA genome of SARS-CoV-2 was purified with TRIzol (Thermo Fisher) from the supernatant of Vero E6 cells infected with SARS-CoV-2, and 1 μg of RNA was reverse transcribed using the iScript cDNA synthesis kit (Bio-Rad). cDNA of the RNA genome of SARS-CoV-2 was used and amplified by PCR through primers with the T7 promoter sequence in the 5′ end for PCR to prepare templates of the in vitro transcription of the 5′ UTR, 3′ UTR, and three other RNA fragments. The purified PCR products were used for genomic RNA fragment synthesis using a HiScribe T7 high-yield RNA synthesis kit (NEB) according to the manufacturer’s instructions. The synthesized genomic RNA fragments were purified and labeled with biotin using the Label IT Biotin Labeling Kit (Mirus) for the RNA pull-down assay and RIP assay with RNA competition. The sequences of primers with the T7 promoter sequence used in this study are listed in *SI Appendix*, Table S1.

### CNBP Peptide Generation.

CNBP peptides were synthesized at GenScript Biotech Corp. These peptides are linked to the HIV TAT protein transduction sequence to facilitate cellular uptake. A lactate dehydrogenase (LDH) assay to determine the cell death was performed using the CytoTox 96® Non-Radioactive Cytotoxicity Assay kit (Promega).

### Mice Infection.

All animal experiments were approved by the Institutional Animal Care and Use Committee at UMass Chan. Animals were kept in a specific pathogen-free (SPF) environment. The *Cnbp* KO mice were generated as described previously (31). *Ifnar* KO mice were obtained from Jonathan Sprent (Scripps). For SARS-CoV-2 infections, 12- to 16-wk-old male and female mice were anesthetized with isoflurane and infected intranasally with 1 × 10^5^ PFUs of SARS-CoV-2 MA 10 strain. Mice were monitored daily for weight loss and survival. Mouse organs were collected at indicated time points and placed in a bead homogenizer tube with 1 mL of DMEM + 2% fetal bovine serum (FBS) for homogenization, and then, 100 μL of this mixture was placed in PBS for tittering or in 300 μL Trizol LS (Invitrogen) for RNA extraction.

### Lung Histology.

Lungs were perfused with 10 U/mL heparin, then intratracheally inflated with 10% buffered-formalin and dissected from mice. Tissues were fixed in 4% PFA overnight and embedded in 10% paraffin. Thin sections (5 µm) were stained by H&E. Histomorphology, grading of histology scores, and evaluation of inflammation of each H&E slide were performed by Applied Pathology Systems.

### Flow Cytometry.

SARS-CoV-2 MA10 virus-infected mice were anesthetized at day 4 postinfection. The mouse lung and spleen were collected and minced in RPMI and filtered through a 70-μm filter, washed and resuspended in red-blood-cell lysis buffer, and then resuspended in MACS buffer. Isolated lung and spleen mononuclear cells were stained with anti-CD64 BV711, anti-CD11b PE, anti-CD45.2, PerCP-Cy5.5, anti-Ly6G FITC, anti-MHCII PE-Cy7, anti-Ly6C antigen presenting cell (APC), anti-Siglec-F AF700, and anti-F4/80 APC-Cy7. The stained cells were washed and resuspended in 4% PFA for 30 min. Cells were acquired on a Cytek Aurora cytometer. Flow cytometry analysis was done with FlowJo software.

### Statistical Analysis.

GraphPad Prism 8 software (GraphPad Software) was used for data analysis using a two-tailed unpaired Student’s *t* test. For mouse in vivo studies, 3 to 16 mice were used per experiment, and Kaplan-Meier survival curves were generated and analyzed for statistical significance. A *P*-value of 0.05 was considered statistically significant (*P < 0.05, **P < 0.01, ***P < 0.001, ****P < 0.0001).

## Supplementary Material

Appendix 01 (PDF)

## Data Availability

All study data are included in the article and/or *SI Appendix*.
