# Supplementary material for: Cellular nucleic acid-binding protein restricts SARS-CoV-2 by regulating interferon and disrupting RNA–protein condensates
Source: Proc Natl Acad Sci U S A. 2023 Nov 14;120(47):e2308355120. doi: 10.1073/pnas.2308355120 (PMC10666094; doi:10.1073/pnas.2308355120)
Supplement: Supplementary file 1 — Appendix 01 (PDF) [file pnas.2308355120.sapp.pdf]

PNAS

Supporting Information for

Cellular nucleic acid-binding protein restricts SARS-CoV-2 by regulating interferon and disrupting RNA-protein condensates

Yongzhi Chen[1]*, Xuqiu Lei[1], Zhaozhao Jiang[1], Fiachra Humphries[1], Krishna Mohan Parsi[2], Nicholas J. Mustone[1], Irene Ramos[3,4], Tinaye Mutetwa[3], Ana Fernandez-Sesma[3], René Maehr[2], Daniel R. Caffrey[5] and Katherine A. Fitzgerald[1]*
[1]Division of Innate Immunity, Department of Medicine, University of Massachusetts Chan Medical School, Worcester, MA, 01605, USA
[2]Program in Molecular Medicine, Diabetes Center of Excellence, University of Massachusetts Chan Medical School, Worcester, MA, 01605, USA
[3]Department of Microbiology, Icahn School of Medicine at Mount Sinai, New York, NY, 10029, USA
[4]Department of Neurology, Icahn School of Medicine at Mount Sinai, New York, NY, 10029, USA
[5]Division of Infectious Diseases and Immunology, Department of Medicine, University of Massachusetts Chan Medical School, Worcester, MA, 01605, USA

*To whom correspondence may be addressed. Email: yongzhi.chen@umassmed.edu or kate.fitzgerald@umassmed.edu

This PDF file includes:

Figures S1 to S6
Tables S1
Supporting text

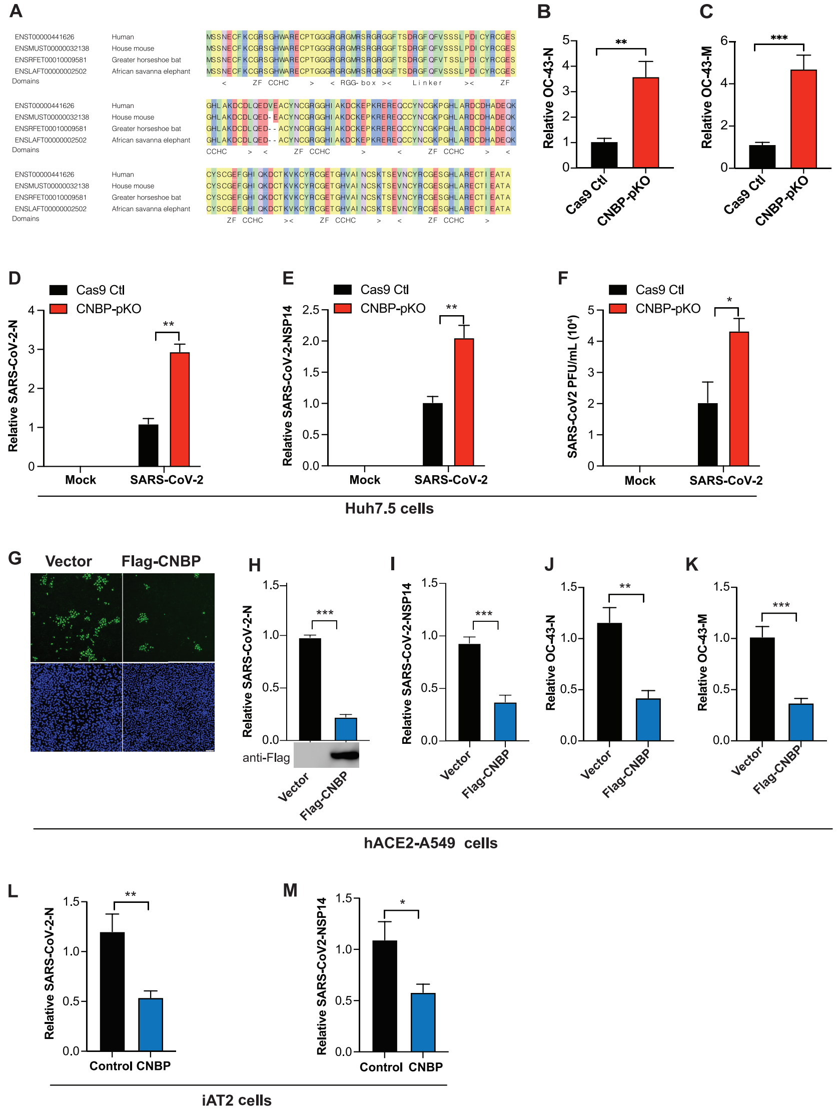

Fig. S1. CNBP inhibits coronavirus OC43 replication in vitro. (A) Multiple sequence alignment of CNBP orthologs from representative lineages in the mammalian phylogeny. The alignment was rendered in PFAAT. Residues are colored by their physicochemical properties and the protein domains are annotated below the alignment. Ensembl IDs and the common name for each species are shown for each ortholog. (B and C) CNBP-pKO and Cas9 Ctl A549 cells were infected with OC43 at an MOI of 0.01. qPCR analysis of viral RNA level of OC43-N (B) and OC43-M (C) at 24 h post-infection. (D–F) Normalized SARS-CoV-2 RNA levels of N (D) and NSP14 (E) as well as the SARS-CoV-2 titers (F) in Huh7.5 WT and KO cells. (G) hACE2-A549 cells were transfected with a Flag-CNBP expression plasmid or control, infected with SARS-CoV-2 for 24 hrs, and dsRNA was visualized by immunofluorescence with anti-J2 antibody (green). Scale Bars, 50 µm (H and I) Normalized SARS-CoV-2 RNA levels of N (H) and NSP14 (I) in hACE2-A549 cells transfected with Flag-CNBP plasmid and infected with SARS-CoV-2. (J and K) Normalized OC43 RNA levels of OC43-N (J) and OC43-M (K) in hACE2-A549 cells transfected with Flag-CNBP plasmid and infected with OC43. (L and M) Normalized SARS-CoV-2 RNA levels of N (L) and NSP14 (M) in iAT2 cells with or without overexpression of CNBP and infected with SARS-CoV-2. Error bars represent SEM of triplicate biological replicates. All data are representative of three independent experiments. *, P<0.05; **, P<0.01; ***, P<0.001.

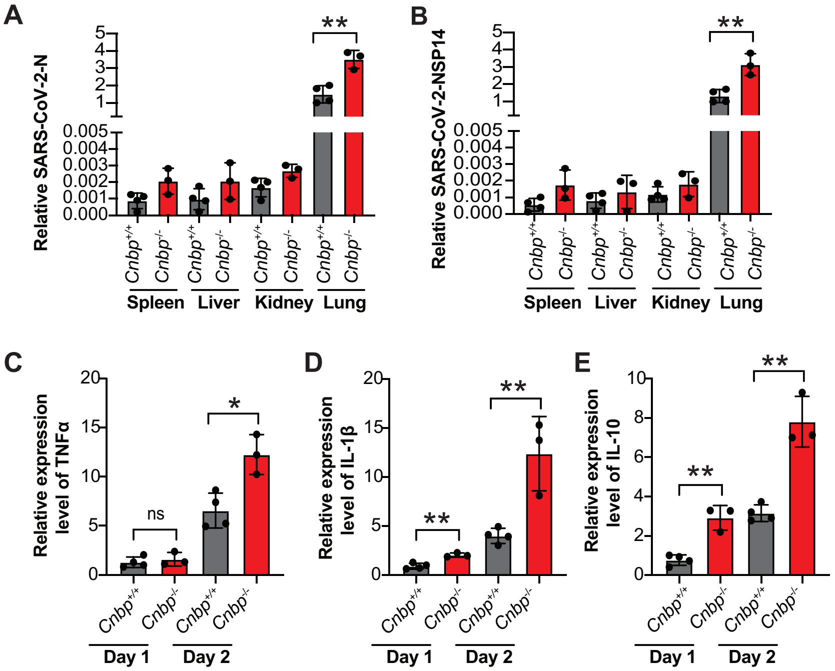

Fig. S2. CNBP inhibits SARS-CoV-2 infection *in vivo*. (A and B) qRT-PCR analysis of SARS-CoV2 virus RNA levels N (A) and NSP14 (B) in variant tissues. (C–E) Normalized mRNA levels of TNFα (C), IL1β (D), and IL-10 (E) from lung samples of mice infected with SARS-CoV-2 MA10 strain. Error bars represent SEM of triplicate biological replicates. All data are representative of three independent experiments. *, $P<0.05$; **, $P<0.01$.

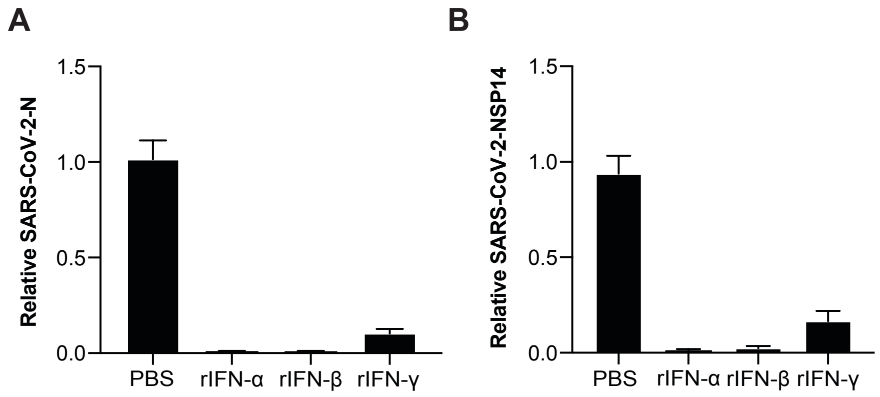

Fig S3. SARS-CoV-2 is sensitive to treatment with IFNs. (A and B) Normalized SARS-CoV-2 RNA levels N (A) and NSP14 (B) in A549-hACE2 cells pretreated with recombinant rIFNa-2b (IFN-α), IFN-β or IFN-γ. Error bars represent SEM of triplicate biological replicates. All data are representative of three independent experiments.

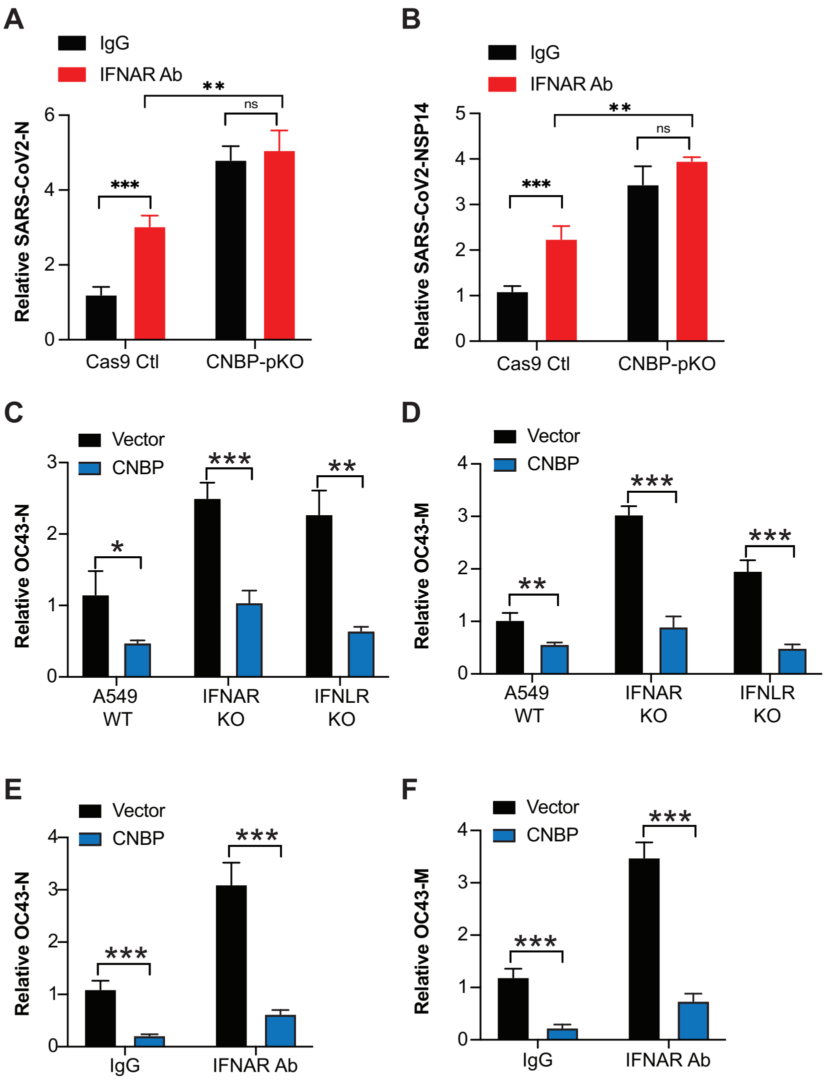

Fig. S4. IFN-independent suppression of OC43 replication by CNBP. (A and B) qRT-PCR analysis of SARS-CoV-2 gRNA expression of N (A) and NSP14 (B) in CNBP pKO and Cas9 control (Ctl) A549 cells treated with the neutralizing antibody anti-IFNAR. (C and D) Normalized OC43 RNA levels of N (C) and M (D) in IFNAR KO, IFNLR KO, and Cas9 Ctl A549 cells transfected with Flag-CNBP. (E and F) Normalized OC43 RNA levels of N (E) and M (F) in A549 cells overexpressing Flag-CNBP treated with neutralizing antibody anti-IFNAR. Error bars represent SEM of triplicate biological replicates. All data are representative of three independent experiments. *, P<0.05; **, *P*<0.01; ***, *P*<0.001.

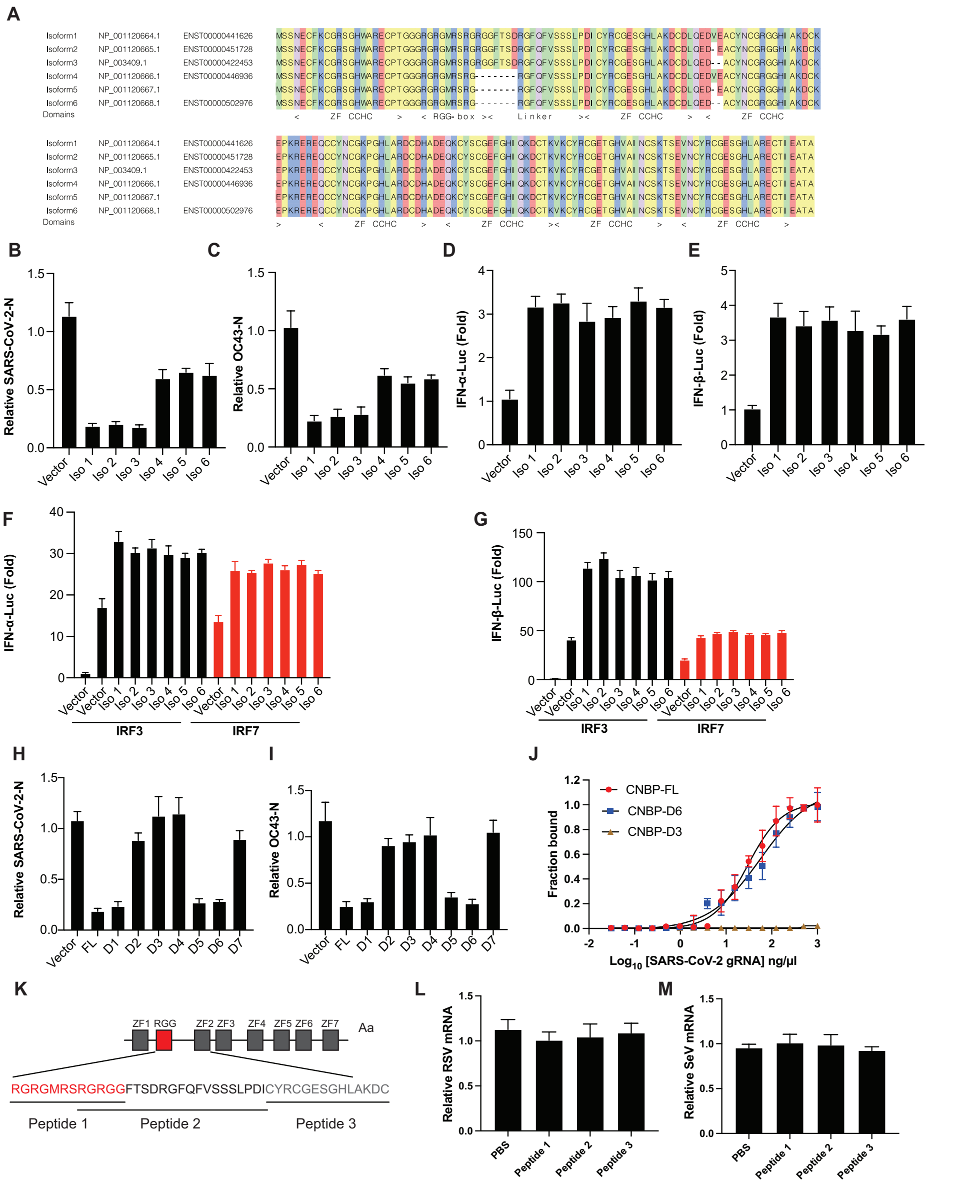

Fig. S5. Analysis of CNBP binding with viral RNA. (A) Multiple sequence alignment of CNBP splice isoforms from human A549 cells. The alignment was rendered in PFAAT. Residues are colored by their physicochemical properties and the protein domains are annotated below the alignment. RefSeq accessions and corresponding Ensembl IDs are shown for each isoform. (B) Analysis of the antiviral function of the six CNBP isoforms on SARS-CoV-2. (C) Analysis of the antiviral function of the six CNBP isoforms on OC43 virus. (D and E) Luciferase activity of IFN-α-Luc (D) or IFN-β-Luc (E) in HEK293 cells after 36-h transfection with the six CNBP isoforms. (F and G) Luciferase activity of IFN-α-Luc (F) or IFN-β-Luc (G) in HEK293 cells after 36h cotransfection of IRF3 or IRF7 with the six CNBP isoforms. (H) Analysis of the antiviral function of the CNBP deletions on SARS-CoV-2. (I) Analysis of the antiviral function of the CNBP deletions on OC43 virus. (J) The RNA binding affinities of CNBP full-length and mutants were measured by MST assays. SARS-CoV-2 genome RNA was assayed in 2-fold concentration steps with RED-Tris-NTA label CNBP full-length or mutants. (K) The sequences of the peptides that mimic CNBP RNA binding regions are shown. (L and M) Analysis of the antiviral function of the CNBP peptides on RSV (L) and SeV (M). Error bars represent SEM of triplicate biological replicates. All data are representative of three independent experiments. **, *P*<0.01.

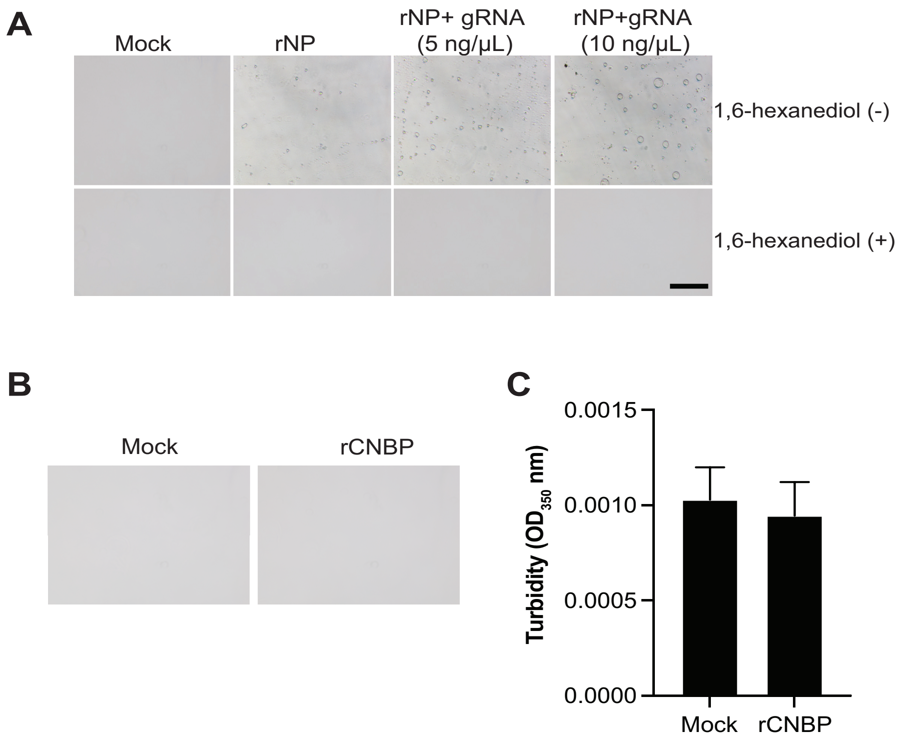

Fig. S6. SARS-CoV-2 N unde-rgoes LLPS. (A) Nucleoprotein LLPS in the presence of SARS-CoV-2 genome RNA observed by bright field using a confocal microscope and disrupted in the presence of 1,6-hexanediol. Scale Bars, 10 µm (B) 20 µM CNBP fails to undergo LLPS. (C) The turbidity of CNBP was measured by absorbance at 350 nm. Error bars represent SEM of triplicate biological replicates. All data are representative of three independent experiments.

Table S1. Primer sequences

Gene	Forward primer	Reverse primer
Q-PCR Primers		
SARS-CoV2-N	CTCTTGTAGATCTGTTCTCTAAACGAAC	GGTCCACCAAACGTAATGCG
SARS-CoV2-Nsp14	TGGGGYTTTACRGGTAACCT	AACRCGCTTAACAAAGCACTC
HCoV-OC43-N	AGGAAGGTCTGCTCCTAATTC	TGCAAAGATGGGGAACTGTGGG
HCoV-OC43-M	GGCTTATGTGGCCCCTTACT	GGCAAATCTGCCCAAGAATA
RSV-1	AACCTTCCACTCAACTTCCTC	GTGATGGGTACTCGGATGTTG
RSV-2	GCTCTTAGCAAAGTCAAGTTGAATGA	TGCTCCGTTGGATGGTGTATT
SeV-1	TGCCTGGAAGATGAGTTAG	GCCTGTTGGTTTGTGGTAAG
SeV-2	AGAGCAGTCCCCTAAGCAGA	TCACCCGGGATCTAGTTGGT
Human IFNb	GTCTCCTCCAAATTGCTCTC	ACAGGAGCTTCTGACACTGA
Human IFNa	CACACAGGCTTCCAGGCATTC	TCTTCAGCACAAAGGACTCATCTG
Human RSAD2	CTTTGTGCTGCCCCTTGAGGAA	CTCTCCCGGATCAGGCTTCCA
Human HPRT	ATCAGACTGAAGAGCTATTGTAATGA	TGGCTTATATCCAACACTTCGTG
murine IFNb	ATAAGCAGCTCCAGCTCCAA	CTGTCTGCTGGTGGAGTTCA
murine TNF-α	GGTGCCTATGTCTCAGCCTCTT	GCCATAGAACTGATGAGAGGGAG
murine IL12b	GGAAGCACGGCAGCAGAATA	AACTTGAGGGAGAAGTAGGAATGG
murine IL10	CGGGAAGACAATAACTGCACCC	CGGTTAGCAGTATGTTGTCCAGC
murine IL1b	CGGCACACCCACCCTG	AAACCGTTTTTCCATCTTCTTCT
murine GAPDH	TGGCAAAGTGGAGATTGTTGCC	AAGATGGTGATGGGCTTCCCG
IVT Primers		
5-UTR	TAATACGACTCACTATAGGGATTAAAGGTTTATACCTTCCCAG	AGAACGTTCCGTGTACCAAGCAA
3-UTR	TAATACGACTCACTATAGGGCAGTAGGGGAACTTCTCCT	TTTTTGTCATTCTCCTAAGAAGCT
5K	TAATACGACTCACTATAGGGCTCCACACGCAAGTTGT	ATTGGTTGCTCTGTGAAATAA
10K	TAATACGACTCACTATAGGGTTCTGATGTTCTTTACCAA	ACCCTTGATTGTTCTTTTCACTGC
20K	TAATACGACTCACTATAGGGTTGATGGTCAAGTAGACTTA	ATCACCAATCAAAGTTGAATCT
sgRNAs		
hCNBP sgRNA1	CACCGCCGTGTGCAGACCCGCGTG	AAACCACGCGGGTCTGCACACGGC
hCNBP sgRNA2	CACCGCGTCCGAGTCTCCGCCGCTG	AAACCAGCGGCGGAGACTCGGACGC
hCNBP sgRNA3	CACCGAAGACGGCTCGCAAGGTAG	AAACCTACCTTGCGAGCCGTCTTC
CNBP PCR clone Primers		
CNBP FL	AAACTCGAGATGAGCAGCAATGAGT	AAAGGATCCGGCTGTAGCCTCAATTGTG
CNBP FL-Mutation	AAACTCGAGATGAGCAGCAATGAGT	AAAGGATCCGGCTGCAGCCTCAATTGCGCATT
CNBP Deletion1	AAACTCGAGATGCGTGGTCGTGGAATGAGAA	AAAGGATCCGGCTGTAGCCTCAATTGTG
CNBP Deletion2	AAACTCGAGATGTCGGATAGAGGTTTCCA	AAAGGATCCGGCTGTAGCCTCAATTGTG
CNBP Deletion3	AAACTCGAGATGGATCTTCAGGAGGATGTTGA	AAAGGATCCGGCTGTAGCCTCAATTGTG
CNBP Deletion4	AAACTCGAGATGAAGGAGCCCAAGAGAGA	AAAGGATCCGGCTGTAGCCTCAATTGTG
CNBP Deletion5	AAACTCGAGATGAGCAGCAATGAGT	AAAGGATCCCAGTCCTTGGCAATGTG
CNBP Deletion6	AAACTCGAGATGAGCAGCAATGAGT	AAAGGATCCACAATCCTTGGCAAGATGAC
CNBP Deletion7	AAACTCGAGATGAGCAGCAATGAGT	AAAGGATCCGGTAAAACCACCTCTG

Supplementary Information Text
Extended Methods:

Cell culture
Human ACE2-A549 cells were a gift from Dr. Benjamin TenOever (NYU Langone Virology Institute), and Vero E6, Huh7.5, or Hek293 cells cultured in Dulbecco's modified Eagle's medium supplemented with 10% (v/v) fetal bovine serum, 100 U/ml penicillin and 100 μg/ml streptomycin.

CRISPR/Cas9 KO
Human ACE2-A549 cells, Huh7.5, or Hek293 cells were seeded on 6-well plates; after 16 h, plasmids expressing Cas9 and single-guide RNA (sgRNA) were co-transfected into cells. At 36 h after transfection, cells were selected for puromycin and blastomycin resistance for another 72–96 hours, then cells were passaged for 1–2 weeks prior to experimental use. Targeting of the desired gene was evaluated by western blot for loss of endogenous protein. sgRNA sequences are shown in Table S1. The generation of CRISPR IFNAR1 KO A549 cells was previously described(1). To generate IFNLR1 KO A549 cells, CRISPR-Cas9 ribonucleoprotein (RNP) complexes (IDT) were transfected using the Nucleofector system (Lonza Bioscience). A predesigned Alt-R CRISPR-Cas9 gRNA targeting exon 3 (design ID: Hs.Cas9.IFNLR1.1.AA), the ATTO 550 Alt-R CRISPR-Cas9 tracrRNA, and the Alt-R S.p. HiFi Cas9 Nuclease were used to form RNPs *in vitro*.

CNBP ortholog analysis
We retrieved CNBP orthologs from the ENSEMBL genome server using the ENSEMBL Compara API (2). We used MUSCLE(3) with default options to align the canonical protein isoforms from *Mus musculus, Rhinolophus ferrumequinum,* and *Loxodonta africana* with the MANE select human isoform. We used PFAAT(4) to annotate the multiple sequence alignment with common species names and protein domains. We rendered the alignment with the Caffrey Wildman residue color scheme to show conservation of physicochemical properties.

CNBP splice isoform analysis
We retrieved Human CNBP splice isoforms from the ENSEMBL genome server using the ENSEMBL API(5). We used MUSCLE(3) with default options to align human CNBP protein isoforms. We used PFAAT(4) to annotate the multiple sequence alignment with protein domains and the linker region that varied across splice isoforms. We used PCR with specific primer pairs to determine which splice variants existed in a cDNA library from human A549 cells.

SI References

1. L. Miorin *et al.*, The oral drug nitazoxanide restricts SARS-CoV-2 infection and attenuates disease pathogenesis in Syrian hamsters. *bioRxiv* 10.1101/2022.02.08.479634 (2022).
2. A. J. Vilella *et al.*, EnsemblCompara GeneTrees: Complete, duplication-aware phylogenetic trees in vertebrates. *Genome Res* **19**, 327-335 (2009).
3. R. C. Edgar, MUSCLE: multiple sequence alignment with high accuracy and high throughput. *Nucleic Acids Res* **32**, 1792-1797 (2004).
4. D. R. Caffrey *et al.*, PFAAT version 2.0: a tool for editing, annotating, and analyzing multiple sequence alignments. *BMC Bioinformatics* **8**, 381 (2007).
5. M. Ruffier *et al.*, Ensembl core software resources: storage and programmatic access for DNA sequence and genome annotation. *Database (Oxford)* **2017** (2017).
